# The Degree of t-System Remodeling Predicts Negative Force-Frequency Relationship and Prolonged Relaxation Time in Failing Human Myocardium

**DOI:** 10.3389/fphys.2020.00182

**Published:** 2020-03-13

**Authors:** Maha Abu-Khousa, Dominik J. Fiegle, Sophie T. Sommer, Ghazali Minabari, Hendrik Milting, Christian Heim, Michael Weyand, Roland Tomasi, Andreas Dendorfer, Tilmann Volk, Thomas Seidel

**Affiliations:** ^1^Institute of Cellular and Molecular Physiology, Friedrich-Alexander-Universität Erlangen-Nürnberg, Erlangen, Germany; ^2^Department of Cardiac Surgery, Friedrich-Alexander-Universität Erlangen-Nürnberg, Erlangen, Germany; ^3^Erich and Hanna Klessmann Institute, Clinic for Thoracic and Cardiovascular Surgery, Heart and Diabetes Center NRW, Ruhr University Bochum, Bad Oeynhausen, Germany; ^4^Muscle Research Center Erlangen (MURCE), Friedrich-Alexander-Universität Erlangen-Nürnberg, Erlangen, Germany; ^5^Walter Brendel Centre of Experimental Medicine, University Hospital, LMU Munich, Munich, Germany; ^6^Department of Anaesthesiology, University Hospital, LMU Munich, Munich, Germany; ^7^German Center for Cardiovascular Research (DZHK), Partner Site Munich Heart Alliance, Munich, Germany

**Keywords:** heart failure, cardiac excitation-contraction coupling, cardiac remodeling, human heart, force frequency relationship, transverse tubular system

## Abstract

The normally positive cardiac force-frequency relationship (FFR) becomes flat or negative in chronic heart failure (HF). Here we explored if remodeling of the cardiomyocyte transverse tubular system (t-system) is associated with alterations in FFR and contractile kinetics in failing human myocardium. Left-ventricular myocardial slices from 13 failing human hearts were mounted into a biomimetic culture setup. Maximum twitch force (F), 90% contraction duration (CD_90_), time to peak force (TTP) and time to relaxation (TTR) were determined at 37°C and 0.2–2 Hz pacing frequency. F_1__Hz_/F_0_._5__Hz_ and F_2__Hz_/F_0_._5__Hz_ served as measures of FFR, intracellular cardiomyocyte t-tubule distance (ΔTT) as measure of t-system remodeling. Protein levels of SERCA2, NCX1, and PLB were quantified by immunoblotting. F_1__Hz_/F_0_._5__Hz_ (*R*^2^ = 0.82) and F_2__Hz_/F_0_._5__Hz_ (*R*^2^ = 0.5) correlated negatively with ΔTT, i.e., samples with severe t-system loss exhibited a negative FFR and reduced myocardial wall tension at high pacing rates. PLB levels also predicted F_1__Hz_/F_0_._5__Hz_, but to a lesser degree (*R*^2^ = 0.49), whereas NCX1 was not correlated (*R*^2^ = 0.02). CD_90_ correlated positively with ΔTT (*R*^2^ = 0.39) and negatively with SERCA2/PLB (*R*^2^ = 0.42), indicating that both the t-system and SERCA activity are important for contraction kinetics. Surprisingly, ΔTT was not associated with TTP (*R*^2^ = 0) but rather with TTR (*R*^2^ = 0.5). This became even more pronounced when interaction with NCX1 expression was added to the model (*R*^2^ = 0.79), suggesting that t-system loss impairs myocardial relaxation especially when NCX1 expression is low. The degree of t-system remodeling predicts FFR inversion and contraction slowing in failing human myocardium. Moreover, together with NCX, the t-system may be important for myocardial relaxation.

## Introduction

The myocardial force-frequency relationship (FFR), also referred to as staircase phenomenon, describes the frequency-dependent change of cardiac contractile force in the absence of other external stimuli and is therefore considered as an intrinsic regulatory mechanism of the heart. Most mammals, including humans, exhibit a positive FFR in the physiological range of heart rates (Janssen and Periasamy, 2007). Because heart rate is increased predominantly at the expense of ventricular filling time, end-diastolic volume decreases at high heart rates. Therefore, systolic force and contraction velocity must increase to maintain a high stroke volume during physical activity (Higginbotham et al., 1986), which, in addition to sympathetic activation, may be facilitated by a positive FFR.

It is widely accepted that dynamic changes in intracellular Ca^2+^ underlie the FFR. At high heart rates, increased influxes of Ca^2+^ and Na^+^ due to more frequent action potentials cause respective rises in intracellular concentrations. This leads to increased Ca^2+^ load of the sarcoplasmic reticulum (SR), because more cytosolic Ca^2+^ is available for the SR Ca^2+^ ATPase (SERCA). Additionally, the increased amount of intracellular Na^+^ reduces the driving force for the sodium-calcium exchanger (NCX). As a consequence, the relative contribution of SERCA to cytosolic Ca^2+^ removal increases. The overall effect is an increase in contractile force. However, while this is one common explanation for the positive FFR in healthy myocardium, additional mechanisms have been suggested (Endoh, 2004; Janssen and Periasamy, 2007), and it remains not completely understood how these mechanisms are altered in diseased myocardium.

The normally positive FFR becomes flat or negative in failing human myocardium (Mulieri et al., 1992). This is thought to contribute to poor cardiac function in heart failure patients, especially under conditions of cardiac stress (Hasenfuss et al., 1994a). It has been shown that altered expression levels and activities of Ca^2+^ cycling proteins, such as SERCA (Hasenfuss et al., 1994b; Munch et al., 2000), PLB (Hasenfuss et al., 1996; Brixius et al., 2003) and NCX (Hasenfuss et al., 1999) are associated with the negative FFR in human failing myocardium.

However, another important requirement for efficient Ca^2+^ cycling and excitation-contraction coupling in ventricular cardiomyocytes is the transverse tubular system (t-system). The t-system is formed by regularly arranged membrane tubules running from the surface into the cytosol, where they form close junctions with the SR. By bringing L-type Ca^2+^ channels close to ryanodine receptors in the SR, Ca^2+^-induced Ca^2+^ release is largely facilitated. Additionally, t-tubules contain a high density of NCX, which may facilitate Ca^2+^ extrusion across the cell membrane (Thomas et al., 2003). A common feature of failing myocardium is remodeling and loss of the t-system (Kostin et al., 1998). The remodeling includes changes in t-tubule structure and reduced density of the t-system and has been associated with poor cardiac function and impaired recovery (Seidel et al., 2017a). Because t-system remodeling impairs intracellular Ca^2+^ cycling and causes slowed as well as reduced Ca^2+^ release (Louch et al., 2004; Seidel et al., 2019), we hypothesized that t-system remodeling may be associated with FFR inversion and altered contractile kinetics.

In this report we explored the correlation of t-system remodeling with the FFR and contraction kinetics in myocardial tissue slices from failing human hearts. We show that high degrees of t-system remodeling are associated with negative FFR and slowed contraction. Furthermore, we show that an interaction between NCX expression and t-system density may predict myocardial relaxation time.

## Method

Methods are described in detail in the Supplementary Material.

### Human Cardiac Samples

Transmural myocardial samples were collected from end-stage failing hearts with reduced ejection fraction from the left-ventricular (LV) apical core during implantation of mechanical assist devices or from the free LV wall of explanted hearts. An overview of the patients is displayed in Supplementary Table S1. Collection and use of human cardiac tissue samples was approved by the Institutional Review Boards of the University of Erlangen-Nürnberg, the Ruhr-University Bochum and the Ludwig-Maximilian University Munich. Studies were conducted according to Declaration of Helsinki principles. Patients gave their written informed consent prior to tissue collection.

### Sample Preparation and Analysis of Contractile Parameters

Following a published method for stable cultivation of human myocardium (Fischer et al., 2019), myocardial slices of 300 μm thickness and approximately 5mm × 5mm length and width were cut with a high-precision vibratome (Leica VT1200S) and installed into biomimetic cultivation chambers at 37°C. Electrodes and a force-transducing wire included in the chambers allowed for continuous electrical stimulation and monitoring of contractile force during auxotonic (elastic) contraction (see Supplementary Figure S1). The sarcomere length at different degrees of preload was assessed in initial experiments by α-actinin staining. Preload was then set to 1.5 mN/mm^2^ in all slices to obtain a resting sarcomere length between 2.0 and 2.1 μm (see Supplementary Figure S2), which is close to the optimum suggested for stable cultivation of myocardial tissue (Watson et al., 2019).

For all cultivated myocardial slices, the baseline stimulation rate was 0.5 Hz. The FFR and single-contraction parameters were assessed within 24 h after installation, using a protocol with intervals of 120 s duration and increasing stimulation frequencies of 0.2, 0.5, 1, and 2 Hz. Contractions during the last 30 s of each interval were analyzed with custom-written software scripts (Matlab 2019a). For each contraction, maximal force (F_max_), time to peak (TTP), time to relaxation (TTR) and 90% contraction duration (CD_90_) were analyzed and then averaged over the 30 s interval. Actively developed wall tension (T) was calculated by dividing the developed force (F) by the cross-sectional area (A): *T* = F/A. To estimate A, the width of the slices (5 mm) was multiplied with the nominal thickness (300 μm) minus 50 μm, assuming that the very top and bottom myocyte layers were damaged during the slicing: *A* = 5mm × 0.25mm = 1.25mm^2^.

### Immunostaining and Confocal Imaging

Neighboring tissue slices of the functionally assessed slices were fixed with 2% PFA immediately after slicing and stained with AF647-conjugated wheat germ agglutinin (WGA, Thermo Fisher, W32466) to visualize the extracellular matrix, surface sarcolemma and t-tubules. RyR2 (Thermo Fisher, MA3-916) or α-actinin (A7811, Sigma) were co-stained with AF488 (A21121, Thermo Fisher). For the validation of the WGA staining, human tissue was co-stained with a mouse monoclonal anti-caveolin-3 antibody (sc-5310, Santa Cruz, CA, United States) and WGA (see Supplementary Figure S3).

The samples were mounted in Fluoromount G (Sigma, F4680) and 3D confocal image stacks (1280 × 1280 × 300 voxels, voxel size 0.1 × 0.1 × 0.1 μm^3^) were acquired with a Zeiss LSM780 confocal microscope with a 63× oil immersion lens. At least 3 image stacks from each sample were recorded from randomly chosen regions. Additionally, two-dimensional confocal tile scans (1–5 mm^2^) were acquired with a pixel size of 0.15 × 0.15 μm^2^. The researchers were blinded against functional parameters.

### Image Analysis

Applying published methods (Seidel et al., 2013, 2016, 2017a,b), confocal image stacks were corrected for depth-dependent attenuation and then filtered, deconvolved and segmented, using histogram-based thresholds. By applying automated analysis scripts, the cardiomyocyte t-system could be distinguished from the outer sarcolemma and interstitial space, using either RyR or α-actinin as cardiomyocyte marker (Seidel et al., 2017a, b). Sarcomere length was determined by 2D Fourier transformation of α-actinin images and identification of the maximum in the power spectrum within a spatial frequency of 1/2.5 to 1/1.5 μm^–1^ (Seidel et al., 2017b). As a measure of t-system remodeling, we calculated the mean intracellular distance to the closest t-tubule (ΔTT), using the three-dimensional Euclidean distance transform of the extracted t-tubule signal and the cardiomyocyte mask (mean ΔTT from at least 3 image stacks per myocardial sample). To assess cardiomyocyte disarray, large area confocal tile scans (1–5 mm^2^) were noise-filtered, deconvolved and then subjected to a watershed-based cardiomyocyte segmentation method published previously (Seidel et al., 2013). In brief, the watershed seeds were generated from the distance transform of the WGA image, followed by a morphological watershed segmentation with the distance transform as gradient image. Subsequently, segments with low contact area to the WGA signal were removed and the watershed run again. This process was iterated until most myocytes contained only one segment. The main axis orientation of myocytes was then determined by calculating the eigenvectors of the covariance matrix (2nd order central image moments) of each segment as described (Lackey et al., 2011).

### Western Blotting

Small transmural tissue blocks from each sample were frozen at −80°C and used later for Western blot analysis. Primary antibodies against SERCA2, NCX1, PLB and phosphorylated PLB (pS16 and pT17) were used. Densitometric measurements were performed with ImageJ/Fiji software and normalized to the geometric means of Ponceau staining and GAPDH. To make samples from different blots comparable, we additionally normalized to a reference, which was added to each gel. A more detailed description of Western blotting, including antibody specifications, is available in the Supplementary Material.

### Statistics

For each myocardial sample, contractile parameters (force, TTR, TTP, CD90, FFR) were assessed simultaneously in 2–8 neighboring tissue slices and the medians used as representative measures for subsequent statistical analyses. Correlation between parameters was tested by linear regression (fitlm function, Matlab version R2019a) with least squares fitting. *P*-values reported for linear models were obtained from an *F*-test against the corresponding constant model (intercept only, i.e., no effect of the predicting variable). *P*-values below 0.05 (type I error) were considered significant. The unpaired two-tailed student’s *t*-test was used to compare means between different groups. The Holm-Bonferroni method was used to correct *P*-values for multiple comparisons. If not indicated otherwise, data are reported as mean ± standard error (SEM).

## Results

### Variable t-System Density and Frequency Response Amongst Myocardial Samples

**FIGURE 1:**
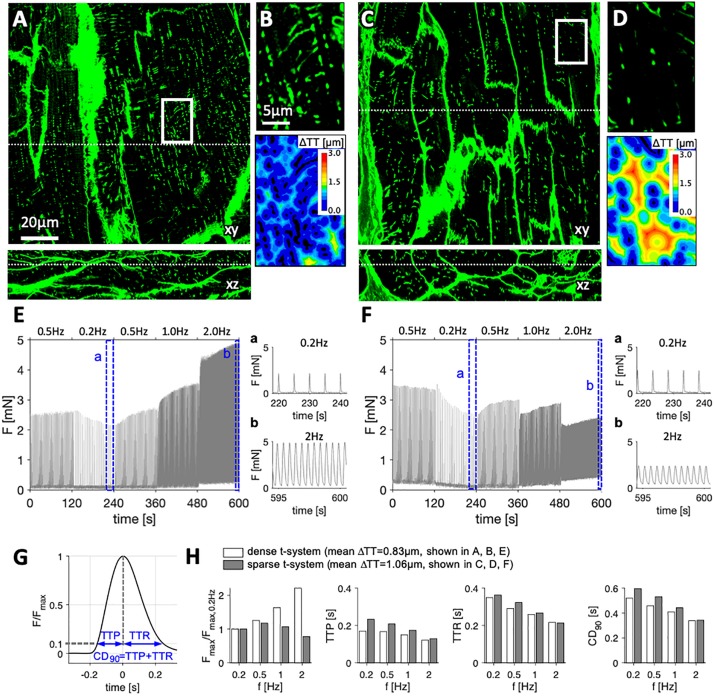
Examples of tissue slices from myocardial specimens with high and low degree of t-system remodeling and corresponding force-frequency relationship. Three-dimensional confocal images in xy and xz views of left-ventricular myocardial tissue slices stained with wheat germ agglutinin from **(A,B)** specimen with dense t-system, and **(C,D)** specimen with sparse t-system. B and D show the respective magnifications of the boxed regions in A and C, and the corresponding 3D distance maps (color-coded distance to closest t-tubule, ΔTT). Dotted lines in A and C indicate the optical sections of the xy and xz views. **(E,F)** Twitch forces of myocardial slices from the specimens shown in A and C, respectively, stimulated with increasing pacing frequency. The highlighted intervals (a, b) are magnified. **(G)** Exemplary trace of one twitch, normalized to its maximum force (F_max_) with schematic of assessed contraction parameters: time to peak (TTP), time to relaxation (TTR) and 90% contraction duration (CD_90_). **(H)** Normalized F_max_, TTP, TTR and CD_90_ at the assessed frequencies in the two examples shown in A (example with low ΔTT, i.e., dense t-system) and in C (example with high ΔTT, i.e., sparse t-system). Scale bar in A also applies to C, scale bar in B also applies to D.

We obtained LV myocardial samples from 13 patients suffering from end-stage heart failure (LV ejection fraction 20.6 ± 7%, Supplementary Table S1) undergoing either LVAD implantation (*n* = 5) or heart transplantation (*n* = 8). In vibratome-cut myocardial slices of 300 μm thickness we assessed the FFR over a frequency range from 0.2 to 2 Hz and stained the extracellular matrix and cell membranes including the t-system with WGA. Reliable staining of t-tubules with WGA was verified by co-staining of caveolin-3, a commonly used membrane marker of cardiomyocytes (Supplementary Figure S3). Three-dimensional confocal microscopic images showed a substantial variability in the degree of t-tubular remodeling between samples from different patients (Figures 1A–D). While some samples presented with a dense and well-preserved t-system and, accordingly, low intracellular t-tubule distances (ΔTT) (Figures 1A,B), others displayed severe t-system loss, resulting in high ΔTT (Figures 1C,D). Inspection of the corresponding FFRs revealed positive relationships in samples with low ΔTT, i.e., preserved t-system (Figure 1E). In these samples the maximum twitch force increased with increasing stimulation frequency. In contrast, samples with high ΔTT, i.e., t-system loss, exhibited a flat response at lower frequencies and a clearly negative response at higher frequencies (Figure 1F). We compared self-normalized forces and the following parameters of contraction kinetics: time to peak force (TTP), time to relaxation (TTR) and 90% contraction duration (CD_90_, Figure 1G). Figure 1H shows that the maximum force in the sample with preserved t-system (low ΔTT) increased more than twofold from 0.2 to 2 Hz, but decreased by approximately 25% in the sample with reduced t-system density (high ΔTT). TTP, TTR, and CD_90_ fitted well to values reported in other studies (Mulieri et al., 1992; Rossman et al., 2004) and decreased consistently in both samples with increasing pacing frequency, but higher values of TTP, TTR, and CD_90_ indicated slowed contraction in the example with t-system loss, especially at low pacing frequencies. In conclusion, the results shown in Figure 1 illustrate that the degree of t-system loss might be related to the FFR and contraction kinetics.

### The Degree of t-System Remodeling Predicts the Degree of FFR Inversion

**FIGURE 2:**
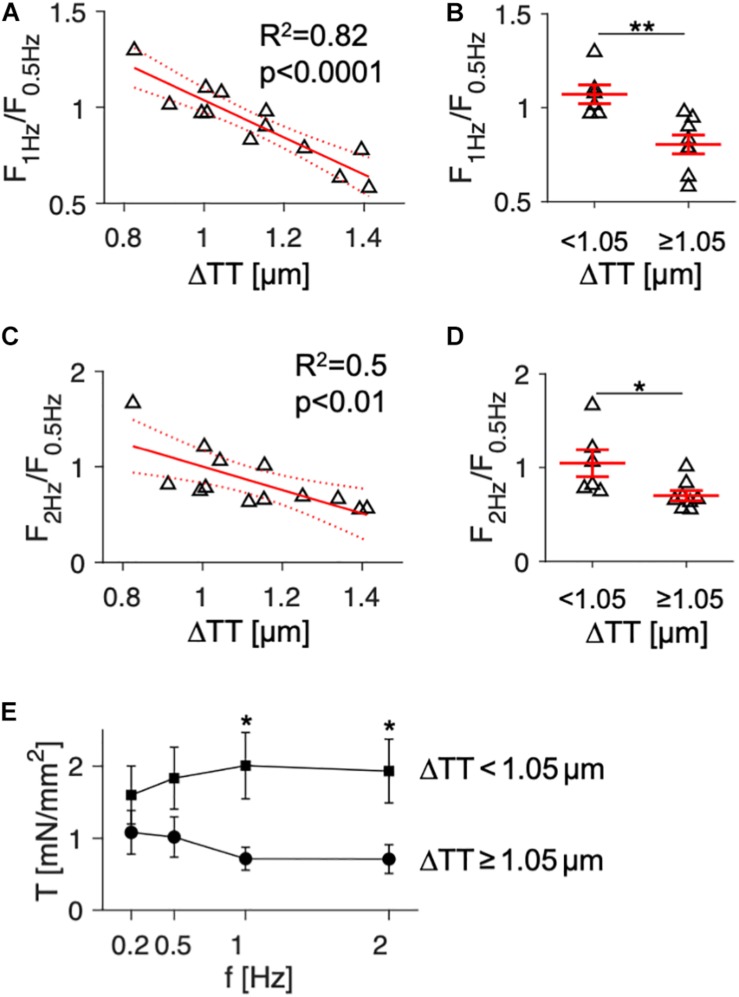
Association of the force-frequency relationship and wall tension with cardiomyocyte t-tubule distance in tissue slices from human failing hearts (*n* = 13). **(A)** Linear model showing the correlation of force at 1 Hz relative to force at 0.5 Hz pacing rate (F_1Hz_/F_0.5Hz_) with t-tubule distance (ΔTT). R^2^ is the coefficient of determination, p indicates the probability of type I error of linear regression. Dotted lines indicate 95% confidence intervals. **(B)** F_1Hz_/F_0.5Hz_ in specimens with low (<1.05 μm, *n* = 7) and high ΔTT (≥ 1.05 μm, *n* = 7). Note that low ΔTT indicates high t-tubule density. **(C)** Correlation of force at 2 Hz relative to force at 0.5 Hz pacing rate (F_2Hz_/F_0.5Hz_) with ΔTT. **(D)** F_2Hz_/F_0.5Hz_ in specimens with low and high ΔTT. **(E)** Mean wall tension (T) developed actively during contraction in specimens with low (squares) and high ΔTT (circles) at 0.2–2 Hz pacing frequency (f). *p < 0.05, **p < 0.01 (unpaired, two-tailed *t*-test, multiple comparison adjustment in E), error bars indicate SEM.

In order to evaluate if ΔTT may generally predict the FFR in failing human myocardium, we performed linear regression including the functional and histological data from all available samples. Mean ΔTT values, acquired from at least 3 confocal image stacks at randomly chosen regions of the tissue slices, ranged from approximately 0.8 to 1.5 μm. This most likely reflects the high regional heterogeneity of t-system loss across the left ventricle in failing hearts, as previously described (Crossman et al., 2015), see discussion. As measures of the FFR, we used the ratio between the maximum force amplitudes at 1 and 0.5 Hz (F_1__Hz_/F_0_._5__Hz_) as well as 2 and 0.5 Hz (F_2__Hz_/F_0_._5__Hz_). Values above or below 1 thus indicate a positive or negative FFR, respectively. Figure 2A shows the linear regression of F_1__Hz_/F_0_._5__Hz_ over ΔTT, which yielded a negative and highly significant relationship between the both parameters. This means that with increasing ΔTT the FFR became more negative (*R*^2^ = 0.82, *p* < 0.0001). The model predicted inversion of the FFR (F_1__Hz_/F_0_._5__Hz_ ≤ 1) at ΔTT ≥ 1.05 μm. Thus, we divided the samples into two groups of similar size: samples with low to moderate t-system loss (ΔTT < 1.05 μm, *n* = 6), and samples with severe t-system loss (ΔTT ≥ 1.05 μm, *n* = 7). We performed a *t*-test to compare the corresponding means of F_1__Hz_/F_0_._5__Hz_ (Figure 2B). Mean F_1__Hz_/F_0_._5__Hz_ in the group with ΔTT < 1.05 μm was 1.08 ± 0.05, indicating a slightly positive FFR, whereas the group with ΔTT ≥ 1.05 μm exhibited a markedly negative FFR (0.80 ± 0.05, *p* < 0.01). When analyzing the relationship between ΔTT and F_2__Hz_/F_0_._5__Hz_ (Figures 2C,D), we found very similar results, although the coefficient of determination was not as high (*R*^2^ = 0.5, *p* < 0.01). In accordance with these findings, mean maximum wall tension did not differ between the two groups at 0.2 and 0.5 Hz pacing frequency, but was distinctively higher at 1 and 2 Hz in the group with ΔTT < 1.05 μm than with ΔTT ≥ 1.05 μm (2.01 ± 0.46 vs. 0.72 ± 0.16 and 1.93 ± 0.44 vs. 0.71 ± 0.2, respectively, in mN/mm^2^, *p* < 0.05) (Figure 2E). In summary, these data indicate that in human failing myocardium a frequency-related increase in contractile force may require a dense t-system and that the degree of t-system remodeling predicts to a substantial extent a functional decline in FFR.

### Association of Ca^2+^ Cycling Proteins With FFR

**FIGURE 3:**
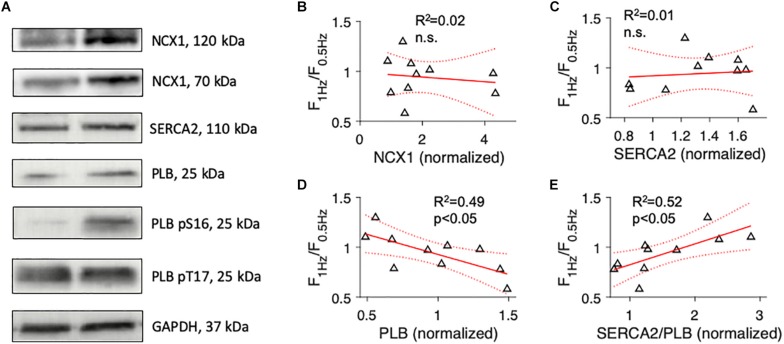
Association of the force-frequency relationship with expression levels of proteins involved in cardiomyocyte Ca^2+^ cycling. **(A)** Example images of Western blots of cardiac sodium-calcium exchanger (NCX1), cardiac sarco/endoplasmic reticulum Ca^2+^-ATPase (SERCA2), total phospholamban (PLB), phospho-S16 (pS16) PLB, phospho-T17 (pT17) PLB, and glyceraldehyde 3-phosphate dehydrogenase (GAPDH). **(B)** Linear model showing the correlation of force at 1 Hz relative to force at 0.5 Hz pacing rate (F_1Hz_/F_0.5Hz_) with NCX1 protein expression (normalized to a reference sample). R^2^ is the coefficient of determination, p indicates the probability of type I error of linear regression. Dotted lines indicate 95% confidence intervals. **(C)** Correlation of F_1Hz_/F_0.5Hz_ with SERCA2 protein expression. **(D)** Correlation of F_1Hz_/F_0.5Hz_ with total PLB protein expression. **(E)** Correlation of F_1Hz_/F_0.5Hz_ with the ratio of SERCA2 to total PLB.

Next, to investigate if Ca^2+^ cycling proteins reported to correlate with the FFR of human failing myocardium predict the FFR to a similar extent as the degree of t-system remodeling, we used Western blotting to quantify protein expression levels of NCX1, SERCA2, and PLB, including its phosphorylated forms pS16 and pT17 (Figure 3A). In 4 out of the 13 samples, the amount of available tissue was not sufficient for protein analysis. Probing for NCX1 yielded clear bands at 120kDa and 70kDa, as described in other studies using the same primary antibody. Because both bands have been shown to represent functional forms of NCX1, we included both in our analyses (McDonald et al., 2000). We found that the expression of Ca^2+^ cycling proteins varied considerably and that the FFR did not correlate with NCX1 or SERCA2 (Figures 3B,C). However, F_1__Hz_/F_0_._5__Hz_ showed a significant negative correlation with total PLB. Samples with negative FFR displayed higher levels of PLB than samples with positive FFR (*R*^2^ = 0.49, *p* < 0.05, Figure 3D). Using the SERCA2/PLB ratio improved the prediction only marginally (*R*^2^ = 0.52, *p* < 0.05, Figure 3E). We also explored whether the extent of PLB inhibition by phosphorylation through PKA (PLBpS16) or CAMKII (PLBpT17) was associated with the FFR. While PLBpS16 and the PLBpS16/PLB ratio did not predict the FFR, the PLBpT17/PLB ratio showed a moderate positive association (*R*^2^ = 0.44, *p* < 0.05, Supplementary Table S2). These results indicate that SERCA2 or NCX1 expression alone were poor predictors of the FFR and that proteins and measures reflecting SERCA2 activity (PLB, SERCA2/PLB ratio, PLBpT17/PLB ratio) predicted the FFR significantly better, but not to the same extent as ΔTT (Figure 2A).

### T-System Loss Is Associated With Slowed Myocardial Relaxation

**FIGURE 4:**
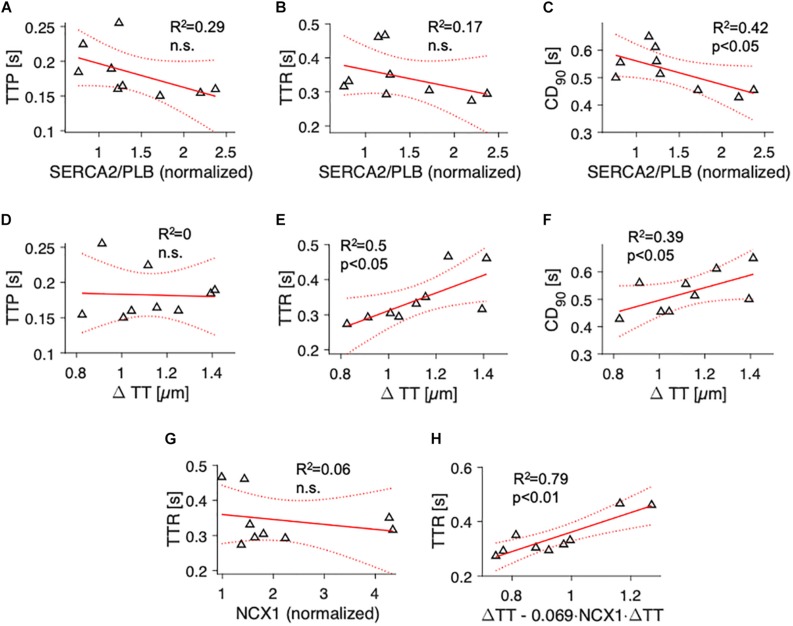
Association of contraction parameters at 1 Hz pacing rate with SERCA2/PLB ratio, t-tubule distance and NCX1 expression. Linear models showing the correlation of **(A)** time to peak (TTP), **(B)** time to relaxation (TTR), and **(C)** 90% contraction duration (CD_90_) with SERCA2/PLB, and of **(D)** TTP, **(E)** TTR and **(F)** CD_90_ with t-tubule distance (ΔTT). R^2^ is the coefficient of determination, p indicates the probability of type I error of linear regression. Dotted lines indicate 95% confidence intervals. **(G)** Correlation of TTR with NCX1. **(H)** Multivariate regression model combining ΔTT and the interaction of ΔTT with NCX (ΔTT + ΔTT:NCX) as predictors of TTR. The fitted model, *TTR = 0.36⋅(ΔTT – 0.069⋅ < *cps*:*it* > *NCX*1 < /*cps*:*it* > ⋅ΔTT)*, explains the variance in TTR significantly better than simple linear models with only NCX1 (*p* < 0.001) or ΔTT (*p* = 0.01, likelihood ratio test).

We then sought to investigate parameters of contractile kinetics. Because contraction kinetics, especially relaxation time, may depend on diastolic sarcomere length (Janssen, 2010), we assessed the degree of cardiomyocyte disarray (Varnava et al., 2001) to rule out that differing fractions of non-aligned myocytes would affect the results (Supplementary Figure S4). Analyzing 2D confocal tile scans of 10 samples, we found that myocyte main axis orientation showed a standard deviation (dispersion) of 18.9 ± 1° and that 11.1 ± 1.8% of the myocytes deviated by more than 30° from the main fiber orientation. Both measures were similar in samples with high and low t-system density (Supplementary Figures S4F,G), allowing the conclusion that myocyte disarray did not bias contraction kinetics. We analyzed individual contractions recorded at 1 Hz stimulation frequency and determined the mean TTP, TTR, and CD_90_ for each sample during a 30 s interval. We then fitted linear models with TTP, TTR or CD_90_ as dependent variables and either SERCA2/PLB (Figures 4A–C) or ΔTT (Figures 4D–F) as predicting parameters. TTP and TTR showed a negative trend with increasing SERCA2/PLB ratio, but statistical significance was reached for CD_90_ only (Figure 4C, *R*^2^ = 0.42, *p* < 0.05). This suggests that the trends on force increase and relaxation time add up and cause a prolonged contraction duration at low SERCA activity. Similar results were obtained for the PLBpT17/PLB ratio (*R*^2^ = 0.49, data not shown). To our surprise, ΔTT did not correlate with TTP (Figure 4D), but rather with TTR and CD_90_ (*R*^2^ = 0.5 and *R*^2^ = 0.39, respectively, *p* < 0.05, Figures 4E,F). This prompted us to test for a relationship between NCX1 and TTR, and interactions between NCX1 and ΔTT, because the velocity of cytosolic Ca^2+^ extrusion via NCX1 may contribute to myocardial relaxation particularly in failing hearts (Hasenfuss et al., 1999). Moreover, NCX1 density has been reported to be high in t-tubules (Thomas et al., 2003). While NCX1 expression was not associated with relaxation time (Figure 4G) or ΔTT (*R*^2^ = 0.1, n.s., data not shown), we found that a multivariate model including ΔTT and the interaction of ΔTT with NCX1 (ΔTT.NCX) predicted TTR significantly better than NCX1 expression or ΔTT alone (*R*^2^ = 0.79, *p* < 0.01, Figure 4H). The data indicate that with increasing degrees of t-system loss contraction duration is prolonged, and that the prolongation is mainly due to an increased relaxation time, which not only depends on the t-system, but also on NCX expression. This can be interpreted as an increased influence of the t-system on relaxation time when NCX expression is low.

## Discussion

A reduced contractile reserve is a hallmark of chronic heart failure and leads to exacerbation of symptoms especially under conditions of cardiac stress, for example during physical activity. While in healthy myocardium, the FFR contributes to adaptation of cardiac output to increased demands, the intrinsic cardiac mechanisms are blunted or even inverted in failing myocardium (Mulieri et al., 1992; Schwinger et al., 1994). Furthermore, impaired myocardial relaxation may add to reduced diastolic filling in certain types of heart failure. Although the cellular and molecular mechanisms leading to these functional impairments have been intensively studied, they are still not entirely clear. It is unknown if remodeling of the t-system, which commonly occurs in chronic heart failure, contributes to FFR inversion and slowing of contraction in intact myocardium from failing human hearts. Here, we describe a close correlation of t-system loss with negative FFR and altered contractile kinetics in human myocardial tissue slices, using confocal microscopy and a recently published method for biomimetic assessment and culture of human myocardium (Fischer et al., 2019). With this, we provide additional evidence that t-system remodeling is an important pathomechanism in heart failure and may predict myocardial dysfunction on a very small spatial scale.

### T-System and FFR

Our results show that there was a high variability in the functional myocardial response to increasing stimulation frequency. This may be surprising, because all patients suffered from end-stage heart failure with severely reduced LV ejection fraction (Supplementary Table S1), and it was shown that the FFR is blunted or reversed on the whole-heart level in heart failure patients (Hasenfuss et al., 1994a). One might therefore expect a negative FFR in all samples. However, considering the finding that myocardial contractility as well as t-system remodeling vary within failing hearts (Crossman et al., 2015), it becomes clear that samples taken randomly from the LV free wall as done in this study, show variations in structure and function. In fact, others also reported high variations in FFR amongst muscle strips obtained from failing hearts (Hasenfuss et al., 1994b). Here, we demonstrate that t-system distance, a histological parameter measuring the degree of t-system loss and remodeling (Seidel et al., 2017a), predicts the FFR with comparable or even higher accuracy than previously reported parameters of protein expression (Hasenfuss et al., 1994b). T-system remodeling may contribute to reduced force development at higher stimulation frequencies by causing decreased and slowed SR Ca^2+^ release due to impaired coupling between L-type Ca^2+^ channels and ryanodine receptors (Louch et al., 2004; Seidel et al., 2019). This may reduce the contractile reserve when fast Ca^2+^ cycling is required.

We also found that high PLB protein expression levels were associated with a negative FFR, suggesting that SERCA inhibition hindered the force increase at high pacing rates. Consistently, a high ratio of PLBpT17 to total PLB, which may reflect SERCA activity, correlated positively with the FFR, whereas SERCA expression levels did not. This is in accordance with a study in failing and non-failing human myocardium reporting a positive correlation of the FFR with SERCA activity, but not with SERCA protein levels (Munch et al., 2000). However, it appears to be in contrast with a study demonstrating that SERCA and PLB overexpression decreased and increased the frequency response, respectively, in isolated rabbit cardiomyocytes (Meyer et al., 1999) and another study reporting that PLB knockout blunts the FFR in mouse hearts (Wu et al., 2012). A possible explanation is that in the cases of SERCA overexpression or PLB knockout SR Ca^2+^ capacity is close to its maximum already at low pacing frequencies and therefore cannot increase at higher frequencies (Janssen and Periasamy, 2007). The degree of PLB phosphorylation at the CAMKII-specific site T17 may reflect CAMKII activity in a sample and therefore correlate positively with the FFR, as CAMKII has been implied as an important modulator of the frequency response (Zhao et al., 2004; Wu et al., 2012). However, considering that CAMKII is activated by cytosolic Ca^2+^, it seems as well possible that increased fractions of PLBpT17 indicate higher intracellular Ca^2+^ levels, which in turn could result from higher t-system density. This becomes clear from the large contribution of the t-system to membrane surface area (Despa et al., 2003; Pasek et al., 2008) and a particularly high density of L-type Ca^2+^ channels in t-tubules (Brette et al., 2006; Seidel et al., 2019). Thus, assuming that L-type Ca^2+^ current density is unaltered or even decreased in samples with low t-system density, a reduction in membrane surface would result in diminished Ca^2+^ influx and might limit the accumulation of intracellular Ca^2+^ required for a positive FFR, especially when the contribution from SR Ca^2+^ is low (Stemmer and Akera, 1986; Endoh, 2004). This mechanism could link t-system density to CAMKII activity, but its investigation would require freezing of the tissue slices immediately after pacing with different frequencies. Here, we quantified PLBpT17 levels at rest only. PLB and the PLBp17/PLB ratio correlated moderately with ΔTT (*R*^2^ = 0.48 and 0.37, respectively). However, dividing the cohort into samples with low and high PLB levels, using the corresponding median as threshold, still yielded high correlations of ΔTT with the FFR (*R*^2^ > 0.7, *p* < 0.05), suggesting that the t-system and resting PLBpT17/PLB levels are independently related to the FFR.

The finding that t-system loss predicts negative FFR, which translates into reduced myocardial wall tension at high pacing rates (Figure 1E), fits well to results from other studies. Acutely detubulated rat myocardium showed a blunted FFR (Ferrantini et al., 2014). Assuming that SERCA and PLB are not affected by detubulation, this may additionally indicate that the dependence of a positive FFR on high t-system density is not confounded by altered SERCA activity in samples with t-system loss. Another study showed that the FFR is flat in human newborns and becomes positive in infants. At the same time, t-tubules develop in ventricular cardiomyocytes (Wiegerinck et al., 2009). Collectively, these findings support the hypothesis that the t-system is required for a positive FFR in human ventricular cardiomyocytes.

### T-System and Contractile Kinetics

In addition to the relationship between the t-system and FFR, we found that t-system loss was associated with slowed contractile kinetics. TTP, however, did not correlate with ΔTT, although acute detubulation was reported to prolong the twitch peak time in rat ventricular trabeculae (Ferrantini et al., 2014). It is possible that due to slower contraction velocity in human than rat hearts (Milani-Nejad and Janssen, 2014), contraction velocity in the rat depends more steeply on the t-system. Moreover, t-system density in rat myocytes is markedly higher than in normal human myocytes (Jayasinghe et al., 2012). T-system loss in rat cardiomyocytes may thus lead to a more pronounced decrease in contraction velocity. Here, a relationship between ΔTT and TTP was not detectable, possibly because of varying age, underlying disease or drug therapy in the samples. Another factor that could affect contractile kinetics is resting sarcomere length (Janssen, 2010). Although we applied a defined diastolic preload to all samples, and found that the degree of myocyte disarray was similar in samples with low and high t-system density (Supplementary Figures S2, S4), we cannot fully exclude that variability stemming from tissue preparation may have masked a possible correlation between ΔTT and TTP. Nevertheless, we did find that ΔTT predicted relaxation time and overall contraction duration. This is consistent with slowed contraction as well as slowed relaxation time observed in acutely detubulated rat myocardium (Ferrantini et al., 2014). As an underlying mechanism we suggest a slowed decay of the intracellular Ca^2+^ transient, because Ca^2+^ removal via NCX is hindered when t-system density is low (Thomas et al., 2003; Ferrantini et al., 2014). This effect may be especially pronounced in human failing myocardium, where the relative contribution of NCX to Ca^2+^ removal seems to be increased because of SERCA downregulation (Hasenfuss et al., 1999). It is well possible that an intact t-system is required for efficient NCX function because the diffusion of intracellular Ca^2+^ ions to the surface sarcolemma may take much longer than to intracellularly located t-tubules. If these are lost, Ca^2+^ extrusion will consequently be slowed, leading to prolonged relaxation time. We provide some evidence for this idea by showing that a linear model taking into account the interaction of ΔTT with NCX predicted the relaxation time significantly better than ΔTT or NCX alone. The negative interaction term in the resulting model points to an increased influence of ΔTT on relaxation time when NCX expression is low. Thus, increased expression levels of NCX found in failing myocardium (Hasenfuss et al., 1996, 1999) might compensate not only for reduced SERCA activity, but also for t-system loss. Based on these exploratory findings we propose a role of the t-system in conjunction with NCX for myocardial relaxation. However, this hypothesis will have to be tested in future studies.

### Limitations

The heart failure patients from whom samples were obtained in this study were heterogeneous regarding etiology, pharmacological treatments and concomitant diseases. Thus, although, the sample size used here (*n* = 13) was comparable to that of other studies (Mulieri et al., 1992; Hasenfuss et al., 1994b; Munch et al., 2000), a larger number of samples would be required to increase statistical power for subgroup analyses, to investigate the influence of clinical parameters on the functional and histological myocardial parameters, and to increase robustness against statistical outliers. However, despite the heterogeneity of the cohort, the main results, that is, correlation of t-system loss with negative FFR and, in conjunction with NCX1 expression, the prediction of slowed myocardial relaxation by t-system loss, showed high statistical significance. This was possibly reached because the structure-function relationships were assessed on a millimeter scale. It remains to be determined if t-system remodeling may predict cardiac function also on larger scales and how t-system remodeling varies across failing hearts.

## Data Availability Statement

The datasets generated for this study are available on request to the corresponding author.

## Ethics Statement

The studies involving human participants were reviewed and approved by the Institutional Review Boards of the University of Erlangen-Nuremberg, the Ruhr-University Bochum and the Ludwig Maximilian University of Munich. The patients/participants provided their written informed consent to participate in this study.

## Author Contributions

TS, TV, AD, CH, MW, and HM contributed to the conception and design. MA-K, DF, SS, GM, RT, and TS contributed to the data acquisition and experiments. TS, MA-K, DF, and RT contributed to the data analysis. TS, TV, and MA-K contributed to the interpretation of the data. MA-K and TS contributed to the drafting of the manuscript. All authors revision and approval of the manuscript.

## Conflict of Interest

The authors declare that the research was conducted in the absence of any commercial or financial relationships that could be construed as a potential conflict of interest.
