# Supplementary material for: The Degree of t-System Remodeling Predicts Negative Force-Frequency Relationship and Prolonged Relaxation Time in Failing Human Myocardium
Source: Front Physiol. 2020 Mar 13;11:182. doi: 10.3389/fphys.2020.00182 (PMC7083140; doi:10.3389/fphys.2020.00182)
Supplement: Supplementary file 1 [file Data_Sheet_1.PDF]

# The degree of t-system remodeling predicts negative force-frequency relationship and prolonged relaxation time in failing human myocardium

## *Supplementary Material*

Maha Abu-Khousa[1], Dominik J. Fiegle[1], Sophie T. Sommer[1], Ghazali Minabari[2], Hendrik Milting[3], Christian Heim[2], Michael Weyand[2], Roland Tomasi[4,5], Andreas Dendorfer[4,6], Tilmann Volk[1,7], Thomas Seidel[1,7]

[1] Institute of Cellular and Molecular Physiology, Friedrich-Alexander-Universität Erlangen-Nürnberg, Erlangen, Germany

[2] Department of Cardiac Surgery, Friedrich-Alexander-Universität Erlangen-Nürnberg, Erlangen, Germany

[3] Erich & Hanna Klessmann Institute, Clinic for Thoracic and Cardiovascular Surgery, Heart and Diabetes Centre NRW, Ruhr-University Bochum, Bad Oeynhausen, Germany

[4] Walter-Brendel-Centre of Experimental Medicine, University Hospital, LMU Munich, Munich, Germany

[5] Department of Anaesthesiology, University Hospital, LMU Munich, Germany

[6] German Center for Cardiovascular Research (DZHK), partner site Munich Heart Alliance, Munich, Germany

[7] Muscle Research Center Erlangen (MURCE), Friedrich-Alexander-Universität Erlangen-Nürnberg, Erlangen, Germany

## 1 Supplementary Data

### 1.1 Western blotting

Cardiac tissue specimens were snap-frozen in liquid nitrogen and stored at -80 °C. For protein extraction they were suspended in TNE-buffer (containing in mmol/L: 50 Tris, 150 NaCl, 1 EDTA, 1 phenylmethylsulfonylfluorid, 1 % Triton X-100 and 0.25 % sodium deoxycholate, pH 7.5) plus phosphatase inhibitor (PhosSTOP, Roche, 4906845001) and mechanically homogenized (T10 basic Ultra Turrax, IKA, Staufen, Germany) on ice for 2x15 s. In addition, cell lysis was completed by sonification (Brandelin, UW70) for 3x5 s with 20 s pauses on ice and repeated vortexing. The lysates were centrifuged for 10 min at 16200×g and the supernatant containing the whole-cell lysate was transferred to a new 1.5 ml reaction tube. For NCX1 protein analysis, 5 mmol/L dithiotreitol was added to TNE buffer. Subsequently, protein concentration was determined by bicinchoninic acid (BCA) assay (Thermo Fisher, 23225) according to the manufacturers' protocol. For Western blotting, protein extracts were loaded on acrylamide gels (see Supplementary Table  for details), separated by electrophoresis for 1.5 h at 25 V and transferred to a PVDF membrane (methanol-activated, 0.45 µm pore size, Carl Roth, Karlsruhe, Germany) with a semi-dry blotting device (Trans Blot SD cell, Bio Rad, Hercules, USA) in blotting buffer (containing in mmol/L: 60 Tris, 50 glycine, 0.0016 SDS, and 20 % V/V methanol) for 45 min at constant voltage (0.25 V/cm$^2$). Total protein quantity was measured using Ponceau S staining solution for 10 min (Fluka BioChemika) and documented by white light

photography on a FusionSL Vilber Lourmat (Vilber, Collegién, France). Ponceau S staining was removed by repeated washings with double-distilled water. Unspecific binding sites were then blocked in blocking solution containing 5 % low-fat milk in Tris-buffered saline with 0.05 % Tween-20 (TBS-T) before staining with the primary antibody. Following three washing steps with TBS-T, the secondary antibody, conjugated to horseradish peroxidase (HRP), was applied. Afterwards, the membrane was washed 3 times. This procedure was similar for all target proteins (see Supplementary Table 3 for details).

Finally, the membrane was incubated for 5 min in the dark with freshly prepared Super Signal West Femto solution (Thermo Fisher, 34096). The chemiluminescence signal was measured with a FusionSL Vilber Lourmat (Volber, Collegién, France) and saturation was avoided by adjusting exposure times of the camera. Dilution series verified that the applied amounts of protein were within the linear detection range of the antibodies and imaging system. For image quantification Fiji/ImageJ software was used (Schindelin et al., 2012). At first, high-frequency noise was removed by Gaussian filtering (sigma=1). Next, local background was subtracted via the rolling ball method (white top-hat transform) with a radius of 50 pixels. This was also applied to photographs of Ponceau S staining. Finally, total signal intensity of bands of the target protein were measured and divided by the sum of signal intensity of several high-intensity Ponceau bands within the same lane, as described elsewhere (Taylor and Posch, 2014; McDonough et al., 2015). One of the tissue samples was randomly selected as reference sample and loaded on every blot which allowed for better comparison between blots (Taylor and Posch, 2014).

# 2 Supplementary Figures and Tables

## 2.1 Supplementary Tables

| | |
|---|---|
| N | 13 |
| age [mean ± SD] | 50.1 ± 11.1 years |
| male / female | 11 / 2 |
| LVAD implantation | 5 |
| HTX | 8 |
| LVEF [mean ± SD] | 20.6 ± 7 % |
| LVEDD [mean ± SD] | 65.7 ± 8.9 mm |
| ICM | 5 |
| NICM | 8 |

**Supplementary Table 1.** Patient characteristics. Abbreviations: N – number of patients, LVAD – left-ventricular assist device, HTX – heart transplantation, NICM – non-ischemic cardiomyopathy, LVEF – left-ventricular ejection fraction, LVEDD – left-ventricular end-diastolic diameter, ICM – ischemic cardiomyopathy

| $F_{1Hz}/F_{0.5Hz}$ ~ | slope | $R^2$ | P |
|---|---|---|---|
| $PLB_{total}$ | negative | 0.51 | 0.02 |
| $PLB_{pS16}$ | positive | 0.002 | 0.91 |
| $PLB_{pT17}$ | positive | 0.24 | 0.15 |
| $PLB_{pS16}$ / $PLB_{total}$ | positive | 0.007 | 0.82 |
| $PLB_{pT17}$ / $PLB_{total}$ | positive | 0.44 | 0.04 |

**Supplementary Table 2.** Linear model parameters from correlation analysis of $F_{1Hz}/F_{0.5Hz}$ with protein expression levels of total ($PLB_{total}$) and phosphorylated forms of phospholamban ($PLB_{pS16}$ and $PLB_{pT17}$). $PLB_{pS16}$ / $PLB_{total}$ and $PLB_{pT17}$ / $PLB_{total}$ indicate the ratio of $PLB_{pS16}$ and $PLB_{pS17}$ to $PLB_{total}$, respectively. n=9; $R^2$, coefficient of determination; P, probability from F-test against constant model

| Target protein | Gel | Protein amount | Primary antibody, Dilution | Secondary antibody, Dilution |
|---|---|---|---|---|
| GAPDH | Depending on target | Depending on target | Thermo Fisher (AM 4300) ,1:20000 | Goat anti-mouse IgG (abcam ab97023), 1:50000 |
| NCX | 8 % | 50 µg | Thermo Fisher (MA3-926), 1:5000 | Goat anti-mouse IgG+IgM-HRP (Thermo Fisher 31446), 1:50000 |
| Phospholamban (PLB total) | 12 % | 10 µg | Thermo Fisher (MA3-922), 1:5000 | Goat anti-mouse IgG (abcam ab97023), 1:50000 |
| Phospholamban (PLB) pS16 | 12 % | 40 µg | Abcam (ab15000), 1:1000 | Goat anti-rabbit (SantaCruz, sc-2054), 1:50000 |
| Phospholamban (PLB) pT17 | 12 % | 40 µg | Badrilla (A010-13AP), 1:500 | Goat anti-rabbit (SantaCruz, sc-2054), 1:50000 |
| SERCA2 | 12 % | 10 µg | ThermoFisher (MA3-919), 1:2000 | Goat anti-mouse IgG (abcam ab97023), 1:50000 |

**Supplementary Table 3.** Target proteins with respective conditions and antibodies used for Western blotting.

## 2.2 Supplementary Figures

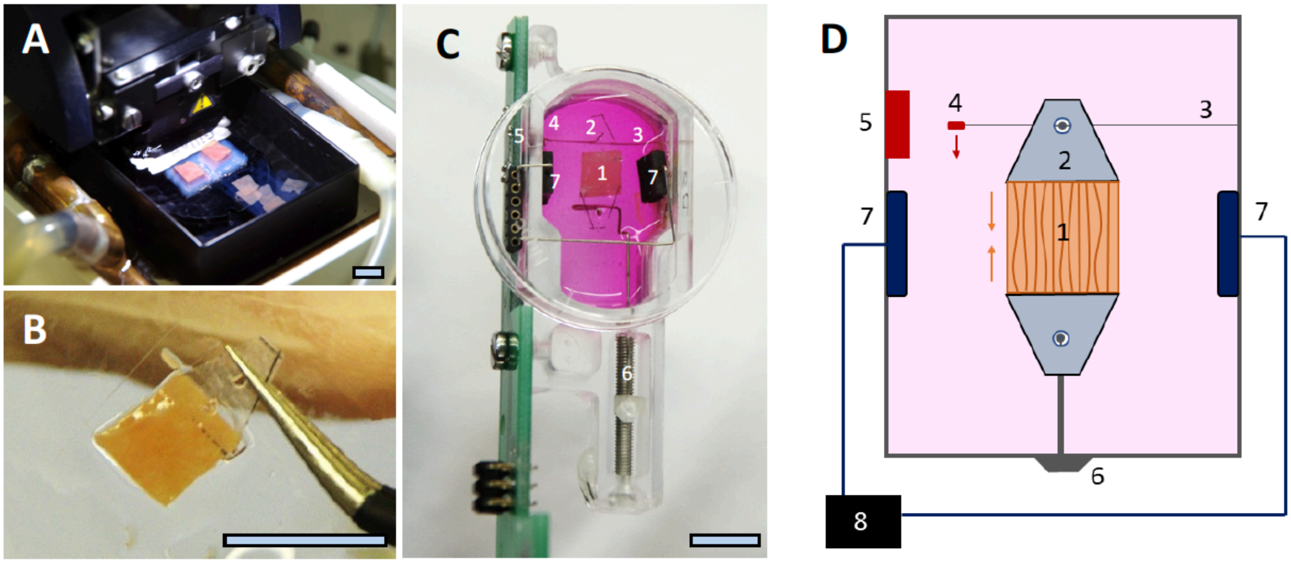


**Supplementary Figure 1.** Experimental workflow and setup for biomimetic cultivation and contraction analysis of human myocardial tissue slices according to a recently published method (Fischer et al., 2019). **(A)** Trimmed and agarose-embedded tissue blocks were submerged into cooled buffer solution and sliced to a thickness of 300 µm. **(B)** Fiber orientation was visually identified, and myocardial tissue slices were further trimmed to a width of 5 mm. Plastic triangles were glued to the opposing ends of the slice, resulting in a tissue area of $5x5mm^2$ available for contraction. **(C)** Detailed view of a slice mounted into a cultivation chamber. **(D)** Schematic illustration. Tissue slices (1) were submerged in culture medium and mounted on steel wires passed through holes in the plastic triangles (2). The flexible wire (3) translated slice contractions into a corresponding deflection with a spring constant of 75 mN/m. The deflection was detected using a small magnet at the tip of the wire (4) and a magnetic field sensor (5). The opposing, non-flexible steel wire was attached to a linear drive (6), which enabled adjustment of the preload. Two graphite electrodes (7) were connected to a pulse generator (8) to allow for electrical field stimulation. Blue scale bars represent 1 cm.

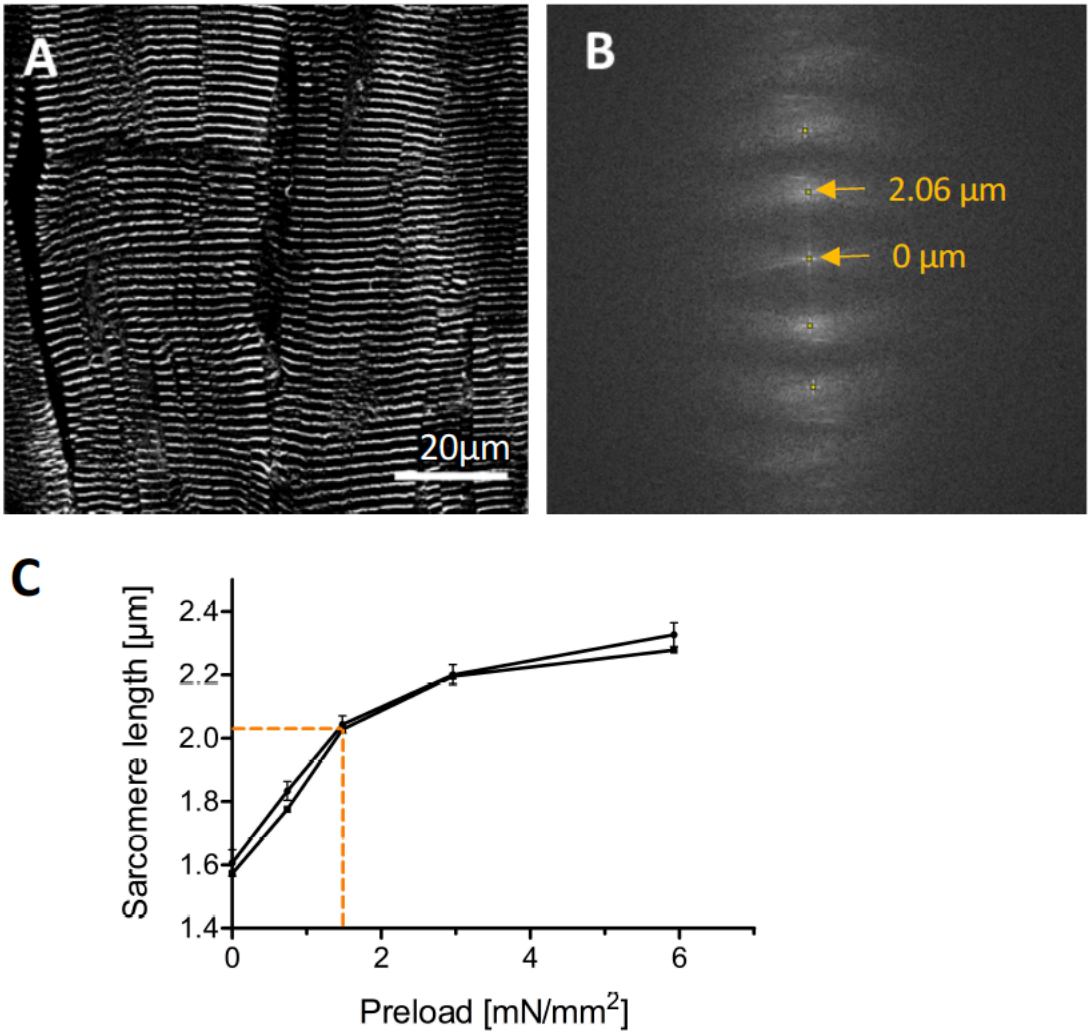


**Supplementary Figure 2.** Relationship between preload and resting sarcomere length. **(A)** Image of a subregion from a two-dimensional confocal tile scan of a human myocardial tissue slice fixed under preload with PFA and stained for α-actinin to visualize the z-disks. The applied preload was 1.5 mN/mm$^2$. **(B)** Two-dimensional Fourier transform of the image shown in A. The maximum of the power spectrum within 1/2.5 and 1/1.5 µm$^{-1}$ was searched. In the shown example, it was detected at a spatial frequency of 0.485 µm$^{-1}$, corresponding to a sarcomere length of 2.06 µm (arrow). **(C)** Preload-sarcomere length relationship from two experiments with 4 different regions analyzed per tissue slice. The preload at a sarcomere length of 2.0-2.1 µm was 1.5 mN/mm$^2$ and subsequently applied to all tissue slices.

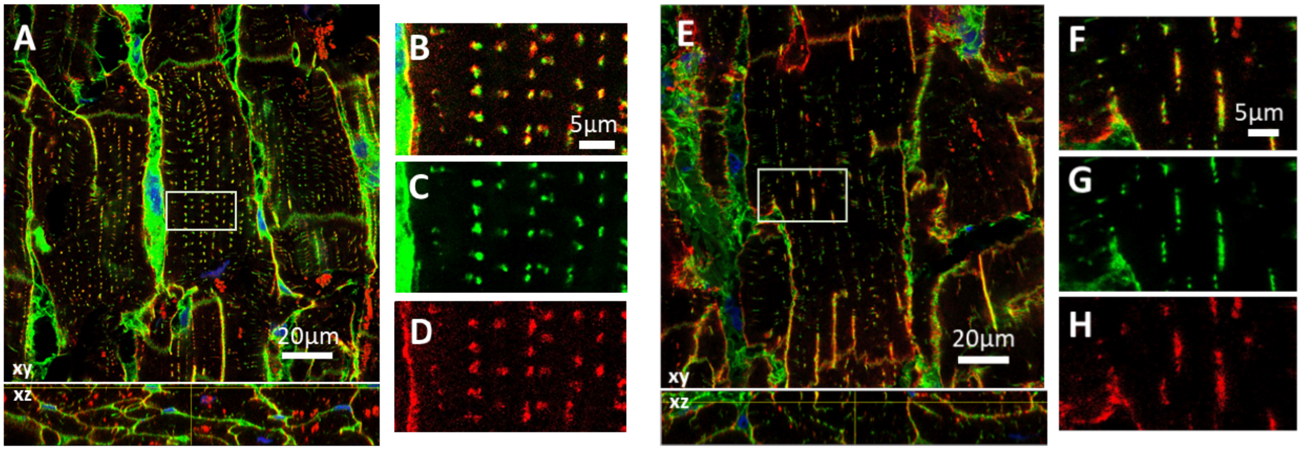


**Supplementary Figure 3.** Three-dimensional confocal images of left-ventricular myocardial tissue slices from failing human hearts co-stained with wheat germ agglutinin (WGA, *green*), antibodies against caveolin-3 (CAV3, *red*), and 4′,6-diamidino-2-phenylindole (DAPI, *blue*). **(A)** XY and XZ views of specimen with high t-system density. **(B-D)** Magnified views of the region highlighted in A. **(E)** XY and XZ views of specimen with low t-system density. **(F-H)** Magnified views of the region highlighted in E. Co-localization of WGA and CAV3 appears *yellow*. Scale bar in A also applies to E. Scale bar in B also applies to C, D, F-H.

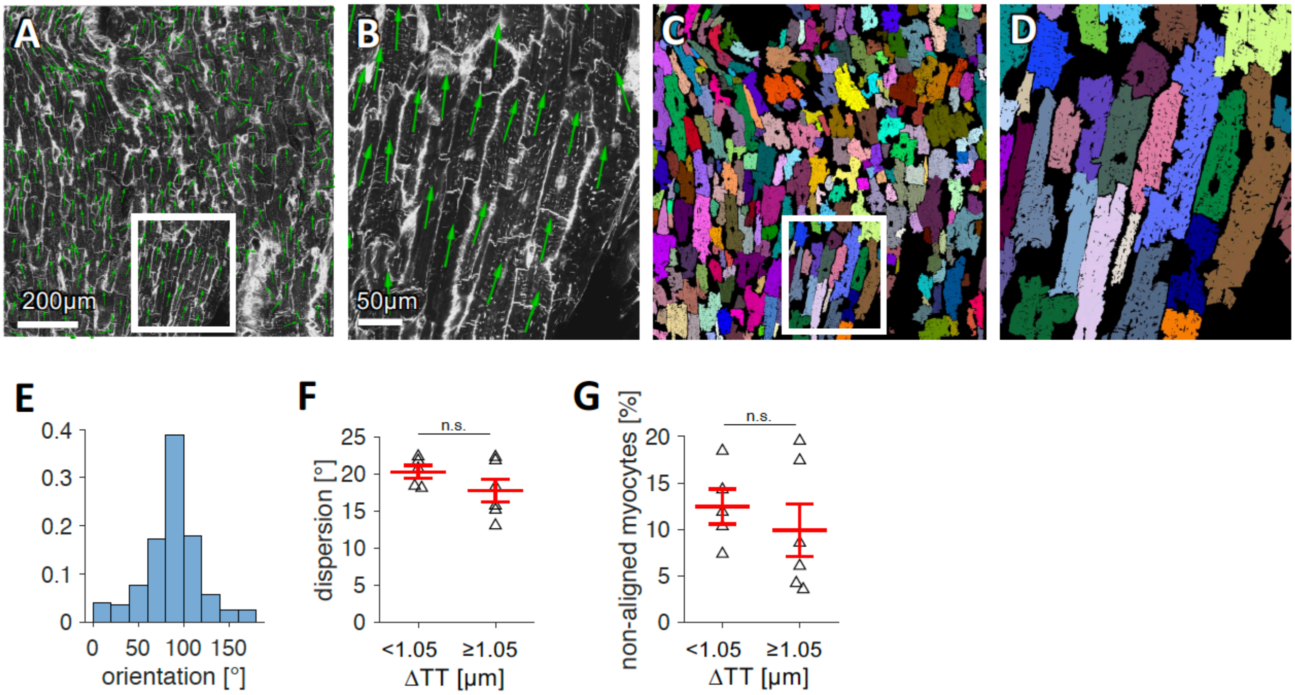


**Supplementary Figure 4.** Assessment of myocyte disarray. **(A)** Two-dimensional confocal tile scan of a myocardial tissue slice stained with wheat germ agglutinin (WGA) for extracellular matrix, cell membranes and t-tubules (scanned area: 1.1x1.1 mm$^2$). **(B)** Magnified view of the region highlighted in A. Green arrows indicate the orientation of the main axis of each segmented myocyte. **(C)** Image showing the segments used for calculating the myocyte orientation. Different myocytes are represented by different colors. **(D)** Magnified view of the region highlighted in C. **(E)** Histogram showing the distribution of myocyte orientation of the example shown in A. The angle was calculated between the horizontal x-axis of the image and the myocyte main axis. **(F)** Myocyte dispersion, defined as the standard deviation of the orientation angles in samples with high (ΔTT<1.05µm) and low t-system density (ΔTT≥1.05µm). **(G)** Percentage of non-aligned cardiomyocytes, defined as myocytes with a main axis deviating more than 30˚ from the mean fiber orientation. Differences between the groups were not significant (n.s., unpaired, two-tailed t-test). Scale bar in A also applies to C. Scale bar in B also applies to D.

## 3 References

Fischer, C., Milting, H., Fein, E., Reiser, E., Lu, K., Seidel, T., et al. (2019). Long-term functional and structural preservation of precision-cut human myocardium under continuous electromechanical stimulation in vitro. *Nat Commun* 10(1)**,** 117. doi: 10.1038/s41467-018-08003-1.

McDonough, A.A., Veiras, L.C., Minas, J.N., and Ralph, D.L. (2015). Considerations when quantitating protein abundance by immunoblot. *Am J Physiol Cell Physiol* 308(6)**,** C426-433. doi: 10.1152/ajpcell.00400.2014.

Schindelin, J., Arganda-Carreras, I., Frise, E., Kaynig, V., Longair, M., Pietzsch, T., et al. (2012). Fiji: an open-source platform for biological-image analysis. *Nat Methods* 9(7)**,** 676-682. doi: 10.1038/nmeth.2019.

Taylor, S.C., and Posch, A. (2014). The design of a quantitative western blot experiment. *Biomed Res Int* 2014**,** 361590. doi: 10.1155/2014/361590.
